# Reducing Chemotherapy-Induced DNA Damage via nAChR-Mediated Redox Reprograming—A New Mechanism for SCLC Chemoresistance Boosted by Nicotine

**DOI:** 10.3390/cancers14092272

**Published:** 2022-05-02

**Authors:** Yuzhi Wang, Tengfei Bian, Lina Song, Yunhan Jiang, Zhiguang Huo, Ramzi G. Salloum, Graham W. Warren, Frederic J. Kaye, Naomi Fujioka, Lingtao Jin, Chengguo Xing

**Affiliations:** 1 Department of Medicinal Chemistry, College of Pharmacy, University of Florida, Gainesville, FL 32610, USA; wangyuzhi@ufl.edu (Y.W.); tbian@cop.ufl.edu (T.B.); 2 Department of Molecular Medicine, UT Health San Antonio, San Antonio, TX 78229, USA; songl1@uthscsa.edu (L.S.); jiangy2@livemail.uthscsa.edu (Y.J.); 3 Department of Biostatistics, College of Medicine, University of Florida, Gainesville, FL 32610, USA; zhuo@ufl.edu; 4 Department of Health Outcomes & Biomedical Informatics, College of Medicine, University of Florida, Gainesville, FL 32610, USA; rsalloum@ufl.edu; 5 Hollings Cancer Center, Medical University of South Carolina, Charleston, SC 29425, USA; warrengw@musc.edu; 6 Department of Medicine, College of Medicine, University of Florida, Gainesville, FL 32610, USA; fkaye@ufl.edu; 7 Department of Medicine, Medical School, University of Minnesota, Minneapolis, MN 55455, USA; fujio002@umn.edu

**Keywords:** SCLC, nicotine, cotinine, platinum-based therapy, DNA damage, chemoresistance

## Abstract

**Simple Summary:**

Tobacco smoking is the dominant risk factor for lung cancer, particularly for small cell lung cancer (SCLC). Smoking is also associated with worse clinical outcomes of SCLC. For SCLC patients, platinum-based chemotherapy (cisplatin or carboplatin), in combination with etoposide, has been the first-line therapy for decades, even with the recent introduction of immunotherapy. One key limitation of such chemotherapy is the quick acquisition of drug resistance. Here, we found that nicotine and its main metabolite, cotinine, reduced the efficacy of chemotherapies to SCLC cells and accelerated drug resistance, which could contribute to poorer survival rates in SCLC patients who continue to smoke.

**Abstract:**

Up to 60% of patients with small cell lung cancer (SCLC) continue to smoke, which is associated with worse clinical outcomes. Platinum-based chemotherapies, in combination with topoisomerase inhibitors, are first-line therapies for SCLC, with rapid chemoresistance as a major barrier. We provided evidence in this study that nicotine and its major metabolite, cotinine, at physiologically relevant concentrations, reduced the efficacy of platinum-based chemotherapies and facilitated chemoresistance in SCLC cells. Mechanistically, nicotine or cotinine reduced chemotherapy-induced DNA damage by modulating cellular redox processes, with nAChRs as the upstream targets. Surprisingly, cisplatin treatment alone also increased the levels of nAChRs in SCLC cells, which served as a self-defense mechanism against platinum-based therapies. These discoveries were confirmed in long-term in vitro and in vivo studies. Collectively, our results depicted a novel and clinically important mechanism of chemoresistance in SCLC treatment: nicotine exposure significantly compromises the efficacy of platinum-based chemotherapies in SCLC treatment by reducing therapy-induced DNA damage and accelerating chemoresistance acquisition. The results also emphasized the urgent need for tobacco cessation and the control of NRT use for SCLC management.

## 1. Introduction

Lung cancer is the leading cause of cancer-related deaths [1,2]. It can be classified mainly into non-small cell lung cancer (NSCLC) and small cell lung cancer (SCLC). With the recent introductions of targeted therapies and immunotherapies, the 5-year survival rate of patients with NSCLC has improved significantly (>20%) [3,4]. The rate for SCLC, however, remains around 5–6% [5,6]. In comparison to NSCLC, SCLC is genetically more heterogeneous, with no known targetable dominant mutations. Thus, there are no targeted therapies for SCLC treatment yet. Because of this, platinum-based chemotherapy (cisplatin or carboplatin), in combination with topoisomerase inhibitors (mostly etoposide), has been the backbone therapy for SCLC patients, even with the recent introduction of checkpoint inhibitors [7]. The mechanism of these chemotherapies is to induce DNA damage. Such DNA damage, if not repaired, trigger cancer cell deaths.

One major challenge for SCLC management is the rapid and common acquisition of chemoresistance [8,9,10,11,12,13]. The same chemotherapies have been widely used for other malignancies, but resistance is not as rapid or universal. This suggests that there might be unique factors among patients with SCLC, contributing to its chemoresistance. One key feature for patients with SCLC is the high prevalence of tobacco use [14,15,16,17,18], especially that up to 60% of them continue to smoke after diagnosis. Although tobacco use is well-known to be associated with worse clinical outcomes among patients with cancer, its impact on SCLC appears to be more dramatic. For instance, a study of 284 patients with SCLC at Mayo Clinic showed that the overall survival was 2.36 years for patients who quit smoking at diagnosis while it was only 1.36 years for those who continued smoking [14]. Although tobacco smoke contains a wide range of chemicals, such as NNK and NNN, nicotine is of much higher abundance. At the same time, nicotine replacement therapy (NRT) has been recommended to patients with SCLC to reduce tobacco exposure [19]. Therefore, nicotine exposure among patients with SCLC is prevalent.

Although nicotine has been largely considered to be safe, non-carcinogenic, and to cause no DNA damage, its oncogenic effects on various malignancies have been reported, including SCLC [20,21,22,23,24,25,26,27,28,29,30,31,32,33,34,35]. Mechanistically, these detrimental effects have been mostly attributed to a range of oncogenic signaling, initiated upon the binding of nicotine to and activation of nicotinic acetylcholine receptors (nAChRs). For instance, nicotine has been reported to stimulate AKT and ERK signaling, which promote cell survival and confer resistance [23,24,25]. Nicotine has also been reported to modulate Bcl-2 family proteins, contributing to chemoresistance [31,32,33,34]. Such oncogenic signaling, however, has not been validated in vivo, particularly in SCLC. Their physiological relevance remains to be determined, particularly given that nicotine has a rather short half-life in smokers (1–2 h), while its peak plasma concentration among smokers is about 15 ng/mL (~0.1 µM) [36]. Cotinine, the major metabolite of nicotine, has a much longer half-life (16 h) and much higher plasma concentration (275 ng/mL, ~1.5 µM) [36]. Mechanistically, cotinine also binds to and activates nAChRs [37], and has been the subject of limited investigations in comparison to nicotine [38].

Given the high prevalence of nicotine exposure among patients with SCLC, this study characterized the impact of nicotine and cotinine on SCLC in vitro and in vivo, particularly with respect to the rapid acquisition of chemoresistance in SCLC treatment.

## 2. Materials and Methods

### 2.1. Cell Lines and Cell Culture Conditions

Human SCLC cell lines used in this paper were obtained from Dr. Lingtao Jin’s lab. They belong to four subtypes: ASCL1 (DMS53, H146, H128, H372, DMS153, H69 and H209), NEUROD1 (H446), YAP1 (H841,DMS114 and H196), and POU2F3 (H1048 and H526). These cell lines were authenticated by genomic short tandem repeat profiling. They were cultured in RPMI 1640 medium supplemented with 10% FBS (Gibco) in a humidified incubator with 5% CO_2_ at 37 °C and tested for mycoplasma monthly.

### 2.2. Cell Viability

Cells were starved for 24 h by culturing in RPMI 1640 medium with 0.5% FBS and seeded in 96-well plates (10,000 or 20,000 cells per well). Cells were treated with chemotherapeutic agents and nicotine/cotinine at the specified concentrations. Cell viability was measured by Cell Titer Blue assay after 2 or 5 days of incubation.

### 2.3. Cell Proliferation

Cells (50,000 cells per well) were seeded in 6-well plates for 12 h with 10% FBS medium and then starved in RPMI 1640 medium with 0.5% FBS for 24 h. Cells were treated with nicotine (10 µM) or cotinine (10 µM) for five days with cell numbers counted.

### 2.4. Western Blotting

Lysates were prepared with RIPA buffer supplemented with a protease inhibitor. Protein concentration was determined with the BCA assay reagent. After SDS-PAGE, proteins were transferred to PVDF membranes, followed by blocking, primary antibody, and secondary antibody incubations. Blots were developed with chemiluminescence Western blotting reagent from Bio-Rad. Detailed information on antibodies can be found in Supplementary Table S1.

### 2.5. Apoptosis Analysis

Cells were treated with chemotherapy, alone or in combination with nicotine or cotinine, for 48 h. Cells were then collected and resuspended in 1× Binding Buffer (1 × 10^6^ cells/mL). Cells suspension (100 µL) was mixed with 5 µL antibody against Annexin V and 5 µL PI and incubated for 20 min, according to the manufacturer’s instructions (Invitrogen, Cat. 88800774 Waltham MA, USA). For γH2A.X and cleaved PARP staining, cells were collected, fixed, and permeabilized with BD Cytofix/Cytoperm Fixation/Permeabilization Solution. Finally, cells were incubated with BD Perm/Wash Buffer (50 µL), with anti-H2AX (5 µL/test), and with anti-Cleaved PARP antibodies, followed by Accuri C6 flow cytometry analysis.

### 2.6. Colony Formation Assay

Cells (2000 cells/well) were seeded in 6-well plates with 10% FBS medium overnight. Cells were then incubated with chemotherapy, nicotine/cotinine, or their combination for 3 days. Cells were then cultured in fresh medium without drugs. Two weeks later, cells were fixed with cold 4% paraformaldehyde and stained with crystal violet dye (0.1% *w*/*v*). The images were taken by Bio-Rad ChemiDoc Imaging system (Version 2.4.0.03) Hercules, CA, USA.

### 2.7. Comet Assay

Comet assay was performed with a commercial kit (R&D SYSTEMS) according to the manufacturer’s instructions. The tail length was calculated by Image J software (Version 1.48) Bethesda, MD, USA.

### 2.8. Tumor Digestion

Fresh tumor tissues were minced and digested in a digestion buffer. With the addition of deoxyribonudease I and collagenase, the sample was incubated at 37 °C for 1.5 h. Cell culture medium with 10% FBS was added to stop digestion. The suspension was filtered through a sterile 70 μm cell strainer. The flow-through sample was centrifuged to collect cell pellet. The cell pellet was suspended in fresh medium with 10% FBS and seeded for culturing.

### 2.9. Quantitative Real-Time PCR (RT-qPCR)

Total RNA was extracted using RNeasy Mini Kit (QIAGEN, Cat. 74106 Hilden, Germany) and reversely transcribed. Quantitative real-time PCR was performed with SYBR Green PCR super mix (BIO-RAD, Cat.1708841 Hercules, CA, USA). Primer sequences are listed in Supplementary Table S2.

### 2.10. GSH, NADPH, and ROS Assays

Intracellular levels of GSH, NAPDH, and ROS were determined by assay kits from Promega.

### 2.11. A 60-Day Exposure of H841 Cells with Nicotine, Cotinine, Cisplatin or Their Combination

H841 cells were treated with cisplatin (1 µM), alone or together with nicotine (10 µM) or cotinine (10 µM), for 3 days. The medium was replaced with fresh medium. Once confluent, cells were treated with the same regimen for 4 more cycles. Then, the concentration of cisplatin was increased by 0.5 µM, with the 5-cycle treatment regimen repeated till the concentration of cisplatin reached 2 µM without obvious toxicity.

### 2.12. In Vivo Xenograft Experiments

NU/J female mice (4–6 weeks of age, *n* = 20) from the Jackson laboratory (Bar Harbor, ME, USA) were maintained in specific pathogen-free facilities, according to animal welfare protocols approved by the Institutional Animal Care and Use Committee at the University of Florida. After 1 week of acclimation, mice were randomized into two groups (control and nicotine groups). Then, 5 × 10^6^ H841 cells, in a mixture of PBS (100 μL) and matrigel (100 μL), were implanted by subcutaneous injection into the right flanks to induce tumor formation. One group was given drinking water and the other group was given nicotine-supplemented drinking water (100 µg/mL) from the same day of tumor implantation. Water consumption was monitored once a week. When the size of the tumor reached 100–150 mm^3^, mice in each group were split into two subgroups, with one subgroup given cisplatin (3.5 mg/kg in 100 µL PBS) twice a week by intraperitoneal (i.p.) injection while the other subgroup was given PBS (100 µL). Bodyweights and tumor sizes were measured twice weekly. Tumor volumes were calculated using (length × width^2^)/2. Upon euthanasia, tumor weights were measured and tumors were stored at −80 °C.

### 2.13. Statistical Analysis

All experiments were repeated at least three times except for the in vivo studies. Student’s *t*-test was used for data analysis with two groups. One-way analysis of variance (ANOVA) was used for data analysis, with no less than three groups, using GraphPad Prism 9. A *p* value ≤ 0.05 was considered statistically significant.

## 3. Results

### 3.1. Nicotine or Cotinine Had No Effect on SCLC Cell Proliferation

**Figure 1:**
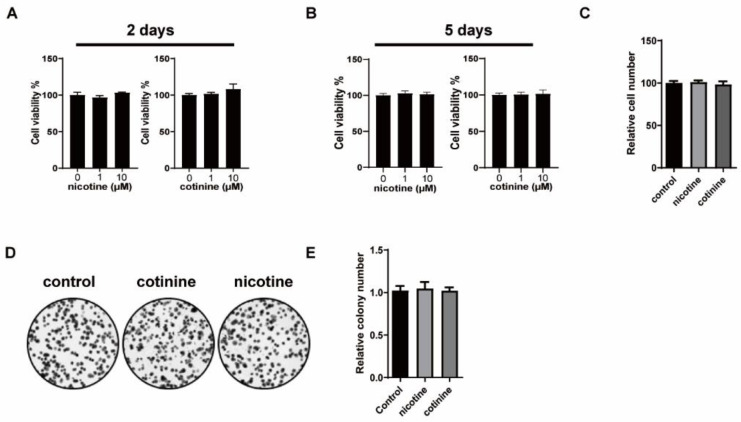
The effects of nicotine or cotinine exposure on H841 cell proliferation. (**A**,**B**) Cell titer blue assay, showing that nicotine/cotinine exposure has no effect on H841 cell viability. H841 cells treated with 1 µM nicotine or 10 µM cotinine for two days (**A**), or five days (**B**), followed by cell titer blue analysis. (**C**) Histogram, showing that nicotine/cotinine exposure has no effect on H841 cell proliferation. H841 cells treated with 10 µM nicotine or 10 µM cotinine for five days and counted. (**D**,**E**) Colony formation analysis, indicating that nicotine/cotinine treatment has no effect on H841 cell proliferation. H841 cells treated with 10 µM nicotine or 10 µM cotinine for 2 weeks, followed by colony formation analysis.

Nicotine and cotinine were evaluated at 1 and 10 µM, which are likely physiologically relevant in the lung tissues, based on their concentrations in smoker blood samples and the direct exposure of lung tissues to tobacco smoke. Two SCLC cell lines (H841 and DMS53) were selected as the model cell lines. Nicotine and cotinine caused no changes on cell viability or proliferation in H841 (Figure 1). Similar results were observed in DMS53 (Supplementary Figure S1).

### 3.2. Nicotine or Cotinine Differentially Reduced the Sensitivity of SCLC Cells to Chemotherapies

**Figure 2:**
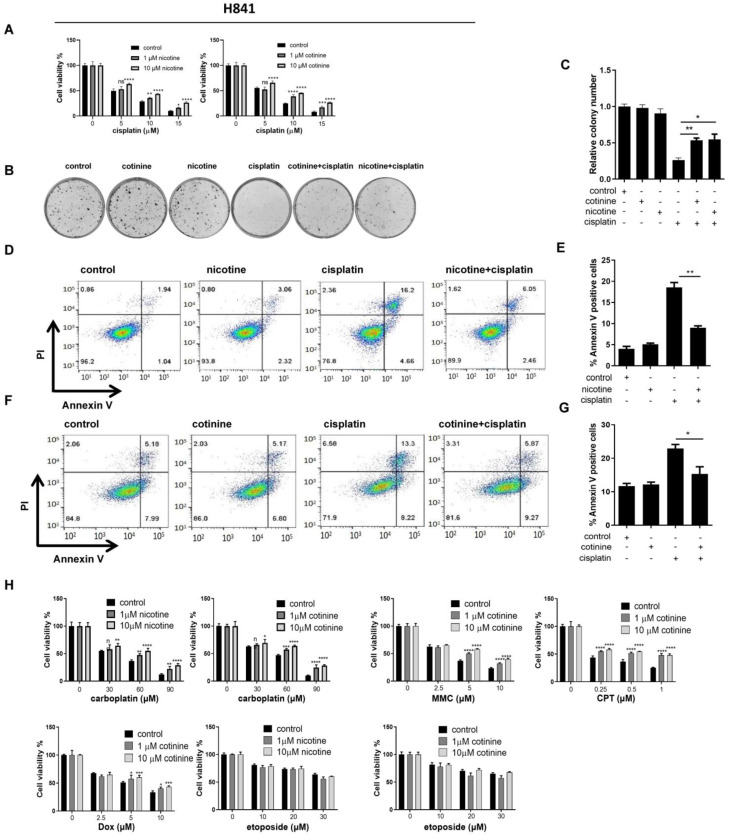
The effects of nicotine and cotinine exposure on the cytotoxicity induced by major SCLC chemotherapies in H841 cells. (**A**) Cell titer blue assay showing that nicotine and cotinine exposure significantly reduce cisplatin-induced SCLC cell cytotoxicity. H841 cells were treated with cisplatin alone, or together with nicotine or cotinine, and cell viability was measured by cell titer blue analysis. (**B**,**C**) Colony formation analysis suggesting that nicotine or cotinine exposure markedly block cisplatin-induced SCLC cell proliferation inhibition. H841 cells were treated with cisplatin alone, or together with 10 µM nicotine or 10 µM cotinine, and colony formation analysis was conducted. (**D**–**G**) Annexin V staining analysis suggesting that nicotine or cotinine exposure significantly suppress cisplatin-induced SCLC cell apoptosis. H841 cells were treated with cisplatin alone, or together with nicotine (**D**) or cotinine (**F**), and cell apoptosis analysis was performed. (**H**), Cell titer blue assay showing that nicotine or cotinine exposure significantly reduce carboplatin, MMC and CPT-induced SCLC cell death, while the effect is weak on Dox and there are no effects on etoposide. H841 cells were treated with DNA damage-inducing reagents, carboplatin, MMC, CPT, Dox and etoposide, alone or together with cotinine, and cell viability analysis was performed. Statistically significant differences determined using *t* test (**E**,**G**) or one-way ANOVA (**H**). *, *p* < 0.05; **, *p* < 0.01; ***, *p* < 0.001 and ****, *p* < 0.0001.

The effects of nicotine or cotinine on the cytotoxicity of chemotherapies to SCLC cells were evaluated, initially with H841 as the model cell line and cisplatin as the model chemotherapy. The reduction in cell viability by cisplatin was significantly attenuated by nicotine and cotinine in a dose-dependent manner (Figure 2A). Similarly, the number of colonies reduced by cisplatin was significantly compromised (Figure 2B,C). Consistently, the numbers of apoptotic cells induced by cisplatin treatment were reduced (Figure 2D–G). We next evaluated the scope of nicotine or cotinine effects on other major chemotherapies used in SCLC treatment, including carboplatin, mitomycin C (MMC), camptothecin (CPT), doxorubicin (Dox) and etoposide in H841 cells (Figure 2H). Significant reductions in cytotoxicity induced by carboplatin, MMC, and CPT were observed by nicotine or cotinine in H841 cells. The reduction in cytotoxicity induced by Dox in H841 by cotinine was relatively weak, while nicotine or cotinine exposure resulted in no reductions in the cytotoxicity induced by etoposide.

We next evaluated the effects of nicotine and cotinine on representative SCLC chemotherapies in a panel of SCLC cell lines. Since SCLC cell lines are classified into four biologically distinct subtypes (ASCL1, NEUROD1, YAP1, and POU2F3) [39], we selected DMS53, H146, H128, H372, DMS153, H69, H209 (ASCL1), H446 (NEUROD1), DMS114, H196 (YAP1), H1048, and H526 (POU2F3) in our study, representing each subtype. For chemotherapies, we focused on cisplatin and etoposide, since they are the first-line chemotherapies for SCLC treatment, and the effects of nicotine and cotinine on these therapies were quite distinct in H841 (Figure 2). The concentrations of nicotine and cotinine remained the same across these cell lines (1 and 10 µM). The concentrations of chemotherapies were selected such that the chemotherapy alone reduced cell viability between 25–75%. Nicotine and cotinine conferred significant protection against cisplatin treatment in DMS53, DMS114, and H446 (Supplementary Figure S2A–C). The same exposure had moderate protection in H128, H69, H209, and H196 (Supplementary Figure S2D–G), with limited effects on H146, H1048, H372, DMS153, and H526 (Supplementary Figure S2H–L). Since nicotine and cotinine failed to reduce the cytotoxicity induced by etoposide in H841 cells, we also evaluated the effects of nicotine and cotinine on etoposide in two additional SCLC cell lines:DMS53 and DMS114. There were no observed effects (Supplementary Figure S2M). Based on our data, in each subtype, nicotine and cotinine exposure caused some cell lines to become less sensitive to cisplatin treatment, except for the subtype POU2F3, which could have been caused by the limited cell lines used in this subtype. Therefore, the effects of nicotine and cotinine do not appear to be SCLC subtype dependent.

Nicotine and cotinine, therefore, could compromise the cytotoxicity of a range of chemotherapies in SCLC cell lines. Additionally, the extent of protection is cell line- and therapy-dependent, potentially related to mechanistic differences among chemotherapies.

### 3.3. Nicotine or Cotinine Reduced Chemotherapy-Induced DNA Damage in SCLC Cells

The common mechanism for the SCLC chemotherapies evaluated above is to induce DNA damage [40,41]. Such DNA damage, if not repaired, will lead to cell death. Although cancer cells utilize a wide range of mechanisms to reduce the cytotoxicity of DNA-damage based chemotherapies, they can be broadly classified as two types of mechanisms: the firstto reduce chemotherapy-induced DNA damage, and the second to reduce cell-death sensitivity to DNA damage. In order to explore how nicotine and cotinine compromised the cytotoxicity of some chemotherapies in SCLC cells, we characterized the effects of such exposure on chemotherapy-induced DNA damage via Western blotting (WB) of γH2A.X, Comet assay [42], and flow cytometry of γH2A.X. Apoptotic status was also characterized by cleaved PARP.

**Figure 3:**
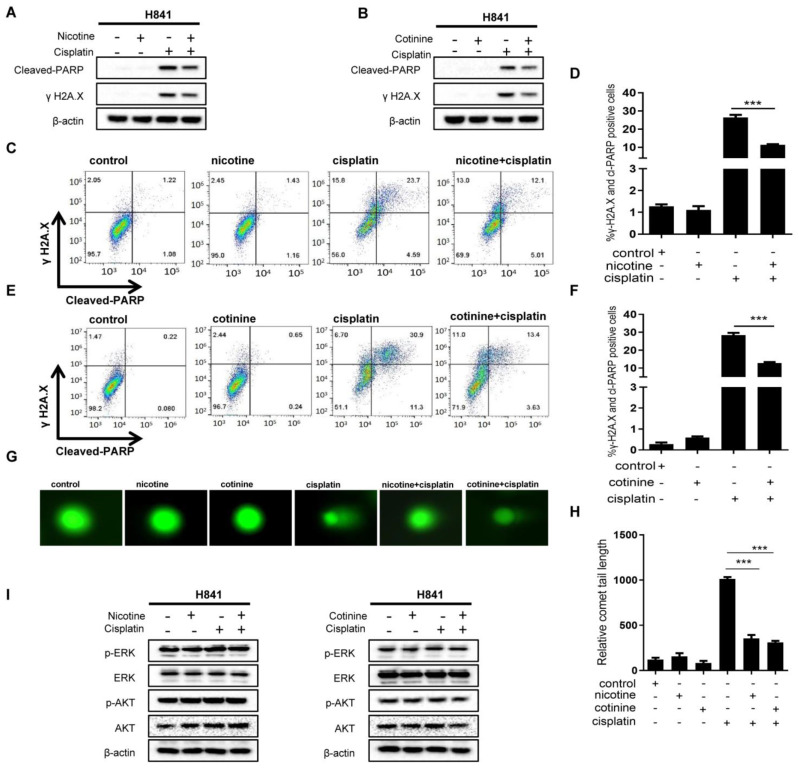
Nicotine or cotinine exposure reduces the sensitivity of H841 to cisplatin by inhibiting cisplatin-induced DNA damage. (**A**,**B**) Western blot analysis suggesting that nicotine or cotinine exposure markedly inhibits cisplatin-induced DNA damage and cell apoptosis. H841 cells treated with cisplatin alone, or together with nicotine (**A**) or cotinine (**B**), and Western blot analysis performed by using antibodies, as indicated. (**C**,**E**) rH2A.X and cleaved-PARP double staining analysis suggesting that nicotine or cotinine exposure significantly inhibits cisplatin-induced DNA damage and cell apoptosis in H841 cells. H841 cells treated with cisplatin, alone or together with nicotine/cotinine, followed by FACS analysis. (**D**,**F**), Histogram showing summary and statistical analysis. (**C**,**E**,**G**) Comet analysis, suggesting that nicotine or cotinine exposure significantly blocks cisplatin-induced DNA damage. (**H**) Histogram showing the statistical analysis in Figure. (**G**,**I**) H841 cells were treated with cisplatin, alone or with nicotine/cotinine, and Western blot analysis performed using antibodies as indicated. Statistically significant differences determined using *t* test (**D**,**F**,**H**). ***, *p* < 0.001. The whole blots could be found in the Figure S6.

H841 was selected as the model cell line, with cisplatin as the model chemotherapy. Cisplatin treatment resulted in marked DNA damage and apoptosis (Figure 3A,B). Such DNA damage and apoptosis were dramatically suppressed by nicotine or cotinine exposure. These effects were also confirmed via flow cytometry analysis of γH2A.X and cleaved-PARP (Figure 3C–F). The results from the Comet assay further supported that nicotine or cotinine exposure reduced cisplatin-induced DNA damage (Figure 3G,H). To define the scope of nicotine or cotinine effects on chemotherapy-induced DNA damage in SCLC cells, we further evaluated their effects on DNA damage induced by MMC, CPT, Dox, and etoposide in H841. Such exposure significantly reduced DNA damage and apoptosis induced by MMC and CPT (Supplementary Figure S3A,B). Moderate reductions were observed with Dox treatment (Supplementary Figure S3C), while such exposure had no effects on DNA damage and apoptosis induced by etoposide (Supplementary Figure S3D). Nicotine or cotinine exposure also had no effects on cisplatin-induced DNA damage in H1048 and H146 (Supplementary Figure S3E,F). The protective effects of nicotine and cotinine against chemotherapies among different SCLC cell lines, therefore, positively correlated with their effects on reducing chemotherapy-induced DNA damage, providing evidence that nicotine or cotinine protected SCLC from chemotherapy treatment.

Our data also argued against the Bcl-2 family protein modulation as the mechanism, which would otherwise confer resistance to all chemotherapies. We also evaluated the effects of nicotine and cotinine on AKT and ERK. Under the conditions that nicotine or cotinine exposure reduced cisplatin-induced DNA damage and apoptosis, no changes were observed on AKT or ERK, including their total abundance and phosphorylated forms in H841 and DMS53 cells (Figure 3I; Supplementary Figure S3G). These results suggested that nicotine or cotinine exposure reduced the sensitivity of H841 to cisplatin by inhibiting cisplatin-induced DNA damage.

### 3.4. Nicotine or Cotinine Modulated Cellular Redox Processes, Contributing to the Reductions in Chemotherapy-Induced DNA Damage in SCLC Cells

**Figure 4:**
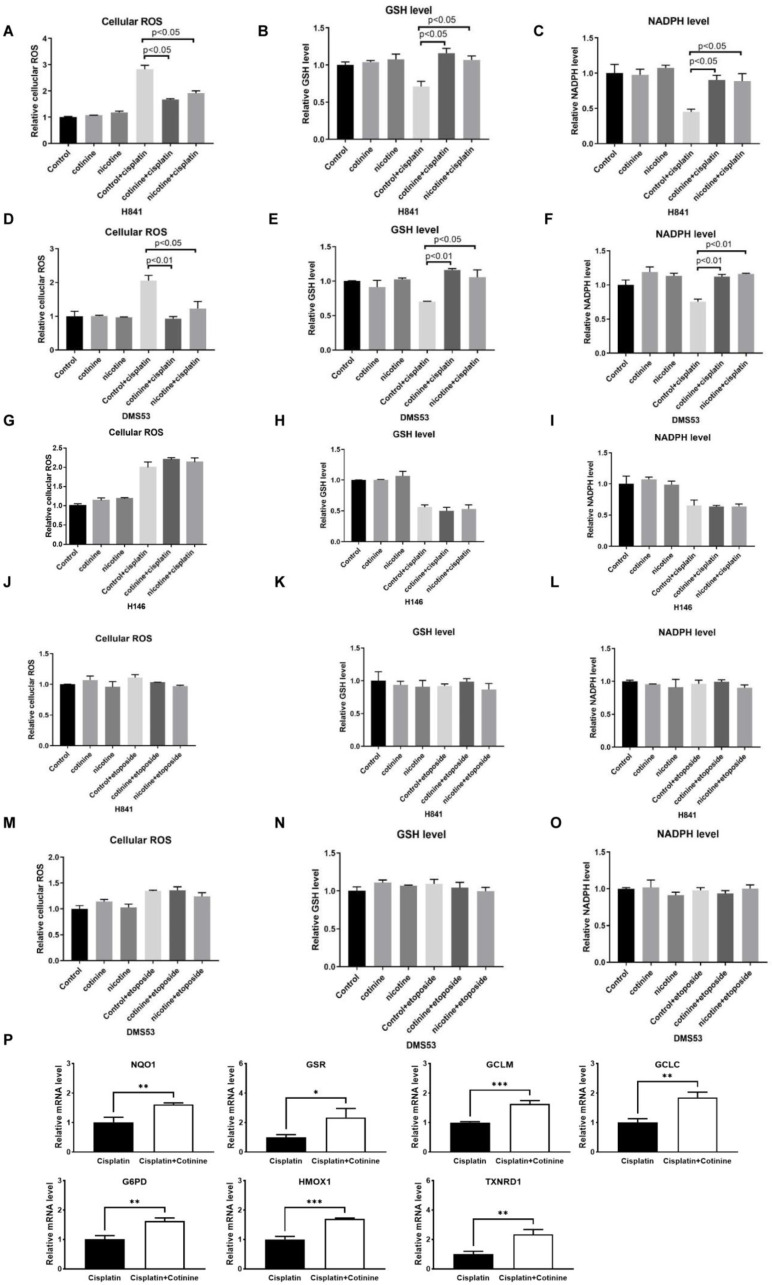
Nicotine- or cotinine-reduced SCLC sensitivity towards cisplatin-induced DNA damage by regulating cellular redox metabolism. (**A**–**C**) Reactive oxygen species (ROS) analysis suggesting that cisplatin-induced cellular ROS could be completely blocked by nicotine or cotinine exposure while cisplatin and nicotine or cotinine co-treatment significantly induces the production of GSH and NADPH in SCLC cells. (**D**–**F**) The effects of nicotine or cotinine on ROS, GSH, and NADPH in DMS53, with or without cisplatin treatment. (**G**–**I**) The effects of nicotine or cotinine on ROS, GSH, and NADPH in H146, with or without cisplatin treatment. (**J**–**O**) The effects of nicotine or cotinine on ROS, GSH, and NADPH in H841 and DMS53, with or without etoposide treatment. (**P**) The effects of cotinine exposure on the expression levels of NRF2 target genes. Statistically significant differences determined using two-tailed student *t* test. *, *p* < 0.05; **, *p* < 0.01; ***, *p* < 0.001.

To explore the mechanisms contributing to nicotine and cotinine’s differential reductions in DNA damage caused by different chemotherapies, we focused on cisplatin and etoposide in H841, DMS53, and H146 cells, given their distinct effects. Cisplatin and etoposide cause DNA damage through different mechanisms; cisplatin reacts with nucleophiles in DNA to form covalent bonds, while etoposide intercalates DNA via non-covalent interactions. Because of their high reactivity, platinum-based therapies can be inactivated by endogenous nucleophiles, including glutathione (GSH) and NADPH [43,44]. Cisplatin also induces oxidative stress, which could increase DNA damage [45,46,47,48,49,50]. We therefore determined whether nicotine or cotinine could affect GSH, NADPH, and cellular reactive oxygen species (ROS). Cisplatin treatment significantly induced cellular ROS. While nicotine or cotinine alone had no effect on cellular ROS, cisplatin-induced cellular ROS were significantly inhibited (Figure 4A–F). Cisplatin treatment also significantly reduced the cellular levels of GSH and NADPH, while nicotine or cotinine exposure offset such reductions (Figure 4A–F). Consistently, nicotine or cotinine treatment showed no effects on the cellular levels of ROS, GSH, or NADPH in the insensitive cell line H146 (Figure 4G–I). Similarly, nicotine or cotinine treatment did not affect their levels with etoposide in both H841 and DMS53 (Figure 4J–O). Given that nAChRs have been reported to regulate cellular redox programing via the NRF2 pathway [51,52,53,54], we analyzed the expression levels of NRF2 target genes known to regulate cellular ROS/GSH/NADPH levels. Cotinine exposure increased the expression levels of several NRF2 target genes involved in GSH metabolism and NADPH generation (Figure 4P). Collectively, these results suggested that cisplatin-induced DNA damage reduction conferred by nicotine or cotinine exposure may be mediated by their ability to reprogram redox homeostasis.

### 3.5. nAChRs as the UpStream Targets and Their UpRegulation upon Platinum-Based Chemotherapy Treatment as a Self-Defense Mechanism for SCLC to Become Less Sensitive to Chemotherapy While Nicotine or Cotinine Accelerate Resistance Acquisition

**Figure 5:**
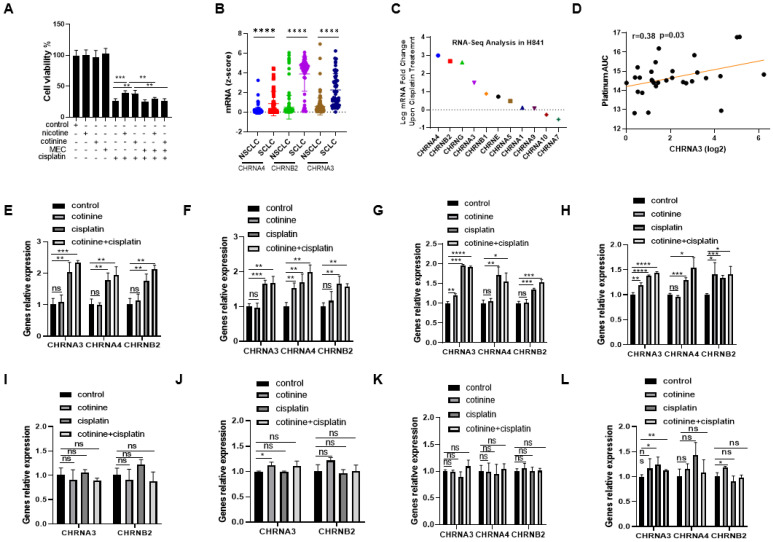
Potential upstream targets for the protective effects of nicotine and cotinine in SCLC against cisplatin treatment. (**A**) The effects of nAChR inhibition (MEC) on nicotine or cotinine-induced cisplatin resistance. (**B**) The levels of CHRNA4, CHRNA3, and CHRNB2 mRNAs in SCLC cell lines, compared to NSCLC cell lines. (**C**) CHRNA4, CHRNA3, and CHRNB2 mRNA levels are upregulated upon cisplatin treatment in H841. (**D**) CHRNA3 mRNA level correlates positively with carboplatin resistance among SCLC cell lines. (**E**) H841, (**F**) DMS53, (**G**) DMS114, and (**H**) H446 exhibit significant increases in CHRNA4, CHRNA3, and CHRNB2 mRNA levels upon cisplatin or cotinine + cisplatin treatment, but with minimal and insignificant changes in (**I**) H1048, (**J**) H146, (**K**) H372, and (**L**) H526 cells. Statistically significant differences determined using *t* test. *, *p* < 0.05; **, *p* < 0.01; ***, *p* < 0.001 and ****, *p* < 0.0001.

We next explored the contribution of nAChRs to the protective effects of nicotine and cotinine. Mecamylamine (a nonselective nAChR antagonist [55]) pretreatment completely blocked the protective effects of nicotine and cotinine against cisplatin in H841 (Figure 5A), supporting nAChRs as the responsible upstream targets. To explore whether certain nAChR isoforms may be more important, we compared the mRNA levels of nAChR isoforms among SCLC and NSCLC cell lines using data from Depmap database [56]. CHRNA4, CHRNB2, and CHRNA3 were significantly higher in SCLC cell lines than NSCLC cell lines [57] (Figure 5B). CHRNA4, CHRNB2, and CHRNA3 were upregulated upon cisplatin treatment in H841 (Figure 5C). Using Cancer Cell Line Encyclopedia [56], we also observed a positive correlation between the mRNA levels of CHRNA3 and insensitivity to carboplatin treatment among SCLC cell lines (Figure 5D) [56]. These data overall supported nAChRs, potentially CHRNA4, CHRNB2 and CHRNA3 isoforms, as responsible for nicotine/cotinine’s protective effects in SCLC cells against chemotherapy treatment. To provide further evidence, the effects of cisplatin on CHRNA4, CHRNB2, and CHRNA3, in the presence/absence of cotinine, were characterized in H841, DMS53, DMS114, and H446 (sensitive to nicotine/cotinine exposure) in comparison to H1048, H146, H372, and H526 (insensitive to nicotine/cotinine exposure). Cisplatin treatment resulted in the upregulation of CHRNA4, CHRNB2, and CHRNA3 mRNA in H841, DMS53, DMS114, and H446 cells (Figure 5E–H) while there was minimal, if any, increase in H1048, H146, H372, or H526 cells (Figure 5I–L). Cotinine co-exposure with cisplatin further upregulated these nAChR isoforms, while cotinine by itself induced minimal effects on these nAChR isoforms in H841, DMS53, DMS114, and H446 (Figure 5E–H). Our data suggested that the upregulation of nAChR may be a general self-defense mechanism for SCLC cells, to reduce the cytotoxic effects of cisplatin and potentially other chemotherapies—while nicotine or cotinine could enhance such an effect.

### 3.6. A 60-Day Nicotine or Cotinine Exposure on H841 Cells Boosted Cisplatin Resistance

**Figure 6:**
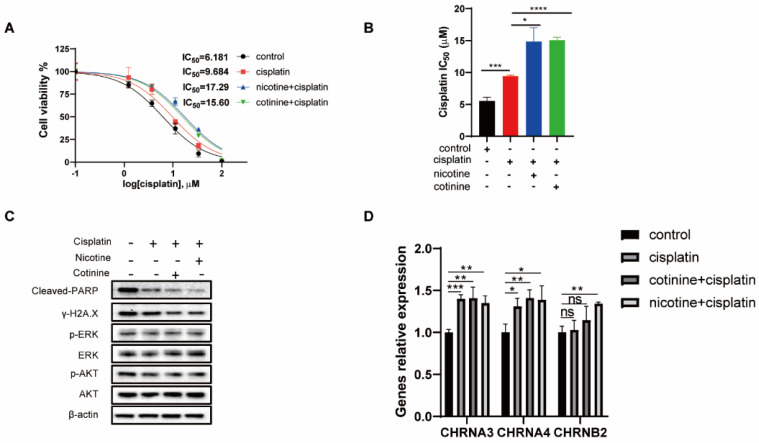
A 60-day exposure of H841 to sublethal cisplatin in the presence or absence of nicotine or cotinine on acquired resistance to cisplatin. (**A**) The dose response curves and IC_50_s of four isogenic H841 cells (continuous exposure of H841 to different treatments for 60 days) to cisplatin treatment. (**B**) Histogram showing the statistical analysis of IC_50_s (*n* = 3). (**C**) After a 60-day chronic exposure to cisplatin, with or without nicotine or cotinine, cells are treated with cisplatin for 48 h and Western blot analysis is performed using antibodies as indicated. (**D**) The levels of CHRNA4, CHRNA3, and CHRNB2 mRNA after a 60-day cisplatin and cotinine/nicotine exposure. Statistically significant differences determined using *t* test. *, *p* < 0.05; **, *p* < 0.01; ***, *p* < 0.001 and ****, *p* < 0.0001. The whole blots could be found in the Figure S6.

To mimic the chronic nature of nicotine/cotinine exposure among SCLC patients, we exposed H841 cells to nicotine or cotinine with/without sublethal dose of cisplatin for 60 days. Such cells were evaluated for their sensitivity towards cisplatin. As expected, H841, upon cisplatin exposure, became resistant towards cisplatin (Figure 6A,B). H841, upon the combinational exposure of cisplatin with nicotine or cotinine, gained even more resistance (Figure 6A,B). No differences were observed on AKT/p-AKT or ERK/p-ERK among these isogenic cells, while the resistant ones had reduced DNA damage (Figure 6C), again supporting the role of DNA damage reduction in conferring cisplatin resistance. H841 cells, upon chronic sublethal dose exposure of cisplatin, also had upregulated CHRNA4, CHRNB2, and CHRNA3, particularly when combined with nicotine or cotinine (Figure 6D). The cisplatin-resistant H841 cells were cross-resistant to carboplatin and MMC (Supplementary Figure S4A,B). No resistances were observed with respect to CPT, Dox, and etoposide treatment (Supplementary Figure S4C–J).

### 3.7. In Vivo Nicotine Exposure on H841-Derived SCLC Tumors Had No Effects on Tumor Growth but Significantly Compromised the Efficacy of Cisplatin Treatment, with Reductions in Cisplatin-Induced DNA Damage

**Figure 7:**
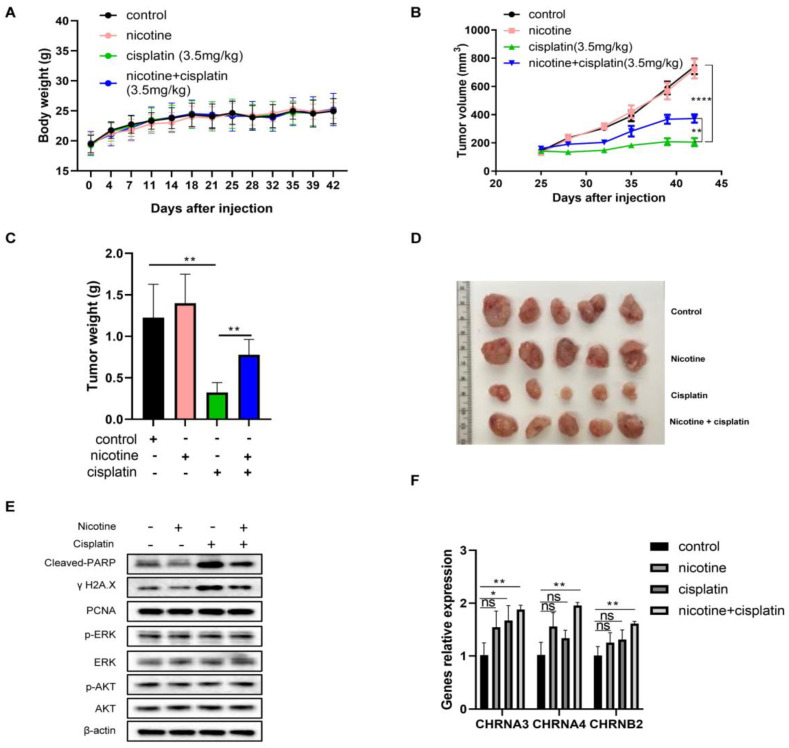
The effects of nicotine on chemoresistance of H841 SCLC tumors to cisplatin in a mouse xenograft model (*n* = 5). (**A**) Body weight changes during the experimental period (measured twice per week). (**B**) Xenograft tumor growth curves. (**C**) The final tumor weights. (**D**) Final tumor images with different treatments. (**E**) Western blotting used to determine the protein levels of p-ERK, ERK, p-AKT, AKT, cleaved-PARP, γ-H2AX, and PCNA with β-actin as the loading control in the combined tumor samples from each group. (**F**) The levels of CHRNA4, CHRNA3, and CHRNB2 mRNA in tumor samples. *t* test used for statistic quantifications: * *p* < 0.05, ** *p* < 0.01, **** *p* < 0.0001, respectively. The whole blots could be found in the Figure S6.

We next assessed the effects of nicotine exposure on SCLC tumors with/without cisplatin in a xenograft model. The amount of daily nicotine intake in the mice (~4 mL water per day) was comparable to the daily nicotine intake among heavy smokers [58,59]. Nicotine and cisplatin were well tolerated, reflected by the similar bodyweight changes among different groups (Figure 7A). Nicotine alone had no effect on tumor growth (Figure 7B–D), consistent with the in vitro results. While cisplatin treatment alone resulted in a significant suppression of tumor growth, nicotine exposure substantially compromised its antitumor efficacy (Figure 7B–D), indicating that nicotine reduced the sensitivity of SCLC cells to cisplatin treatment. Cisplatin-induced DNA damage and apoptosis in residue tumor tissues were substantially reduced with nicotine exposure, while the proliferation markers remained the same (Figure 7E and Supplementary Figure S5A). Similarly, there were no differences in AKT and ERK among different treatments. The levels of mRNAs of CHRNA4, CHRNB2, and CHRNA3 in the tumor tissues were also quantified. Nicotine exposure alone and cisplatin treatment alone appeared to increase the mRNAs of CHRNA4, CHRNB2, and CHRNA3 in the tumor samples, while their combination resulted in further increases (Figure 7F). Lastly, the fresh tumor samples were digested into single cells and cultured to evaluate their sensitivity towards cisplatin via cell viability assay, during which no nicotine or cotinine was added (Supplementary Figure S5B). The results suggested that tumor cells with nicotine exposure alone did not change their sensitivity to cisplatin (IC_50_ = 7.00 µM with nicotine exposure vs. IC_50_ = 6.85 µM for the control) while tumor cells from mice with cisplatin exposure had some reduced sensitivity (IC_50_ = 7.41 µM with cisplatin exposure). Tumor cells from mice with the combination of nicotine and cisplatin exposure resulted in the lowest sensitivity to cisplatin (IC_50_ = 8.22 µM with nicotine/cisplatin co-exposure).

## 4. Discussion

Chemoresistance is a major challenge in SCLC treatment. This work, for the first time, demonstrated that nicotine and cotinine exposure differentially reduced the sensitivity of SCLC cells to key SCLC chemotherapies. Such resistance was mediated through the direct reduction in chemotherapy-induced DNA damage. Mechanistically, cisplatin treatment resulted in significant increases in cellular ROS level and reductions in GSH and NADPH in SCLC cells. Nicotine/cotinine exposure restored their levels in nicotine/cotinine sensitive cells, but not the insensitive cell line. Etoposide treatment, however, had no effects on the cellular levels of ROS, GSH, and NADPH, which could explain the lack of effects of nicotine/cotinine exposure on its sensitivity. We further demonstrated that these protective effects were mediated through nAChRs. To our surprise, nicotine/cotinine sensitive SCLC cells upregulated nAChRs upon cisplatin treatment alone, while cisplatin treatment could not upregulate nAChRs in the nicotine/cotinine-insensitive SCLC cells. These results suggested that nAChR upregulation could be a self-defense mechanism for some SCLC cells to counteract the anticancer activity of platinum-based chemotherapies through the modulation of cellular redox processes that may deactivate chemotherapies. The combination of nicotine/cotinine with platinum-based chemotherapies, which happens when patients with SCLC use tobacco products or NRT during their treatment cycles, offers SCLC cells survival advantage, resulting in quicker chemoresistance and worse clinical outcomes. Further study is needed, particularly in clinical settings, to determine the potential impact on our patients with SCLC and how to reduce nicotine exposure among our patients.

## 5. Conclusions

The current study found that nicotine and cotinine exposure compromised the efficacy of cisplatin and several other key chemotherapies for SCLC treatment through a unique mechanism of action. Combined with prior research examining the role of tobacco and nicotine use in cancer treatment, the current findings have clinical significance to improve SCLC management. First of all, for SCLC patients who smoke at time of diagnosis, tobacco and nicotine cessation support should be included as part of their evidence-based treatment regimen [60]. In addition, these results also emphasized the importance of smoking status as a variable in tailoring treatment regimens for SCLC patients. Importantly, future clinical studies to optimize SCLC management should develop methods and strategies based on the patient’s current or previous tobacco and nicotine use status [61].

## Data Availability

The data presented in this study are available in this article (and Supplementary Material).

## Supplementary Materials

The following supporting information can be downloaded at: https://www.mdpi.com/article/10.3390/cancers14092272/s1, Figure S1: Nicotine or cotinine exposure has no effect on DMS53 cell proliferation; Figure S2: The effects of nicotine or cotinine exposure on the cytotoxicity of cisplatin and etoposide among different SCLC cells; Figure S3: The effects of nicotine or cotinine on different chemotherapeutic agents exposure among different SCLC cells with respect to DNA damage and cell apoptosis; Figure S4: Cross-resistance of cisplatin-resistant H841 isogenic cell lines to other therapies; Figure S5: Nicotine enhances chemoresistance to cisplatin in a mouse xenograft model; Figure S6: The whole blots; Table S1: Antibodies used for WB; Table S2: Primers used for qPCR.

## Abbreviations

SCLC	small cell lung cancer
NSCLC	non-small cell lung cancer
NRT	nicotine replacement therapy
nAChRs	nicotinic acetylcholine receptors
EMT	epithelial to mesenchymal transition
GSH	glutathione
NADPH	nicotinamide adenine dinucleotide phosphate
ROS	reactive oxygen species
MMC	mitomycin C
CPT	camptothecin
Dox	doxorubicin
FACS	fluorescence-activated cell sorting
