# Supplementary material for: Reducing Chemotherapy-Induced DNA Damage via nAChR-Mediated Redox Reprograming—A New Mechanism for SCLC Chemoresistance Boosted by Nicotine
Source: Cancers (Basel). 2022 May 2;14(9):2272. doi: 10.3390/cancers14092272 (PMC9100082; doi:10.3390/cancers14092272)
Supplement: Supplementary file 1 [file cancers-14-02272-s001.zip › cancers-1660914-supplementary.pdf]

Table S1. Antibodies used for WB.

Antibodies	Company	Catalog number
P-ERK1/2 (T202/Y204)	Cell Signaling Technology	9101S
ERK1/2	Cell Signaling Technology	9102S
P-AKT (S473)	Cell Signaling Technology	4060S
AKT	Cell Signaling Technology	9272S
Phospho-Histone H2A.X (Ser139)	Invitrogen	VH307705
PCNA (PC10)	Cell Signaling Technology	2586
Cleaved PARP (Asp214) (D64E10)	Cell Signaling Technology	5625
Anti-mouse IgG, HRP-linked Antibody	Cell Signaling Technology	7076
Anti-rabbit IgG, HRP-linked Antibody	Cell Signaling Technology	7074
Mouse monoclonal anti-beta-actin	Sigma Aldrich	A2228

Table S2. Primers used for qPCR.

Gene name	Sequence (5’-3’)	
	Forward	Reverse
CHRNA3	GGTGGACGACAAGACCAAAG	GGGAAGTAGGTCACGTCGATT
CHRNA4	GGAGGGCGTCCAGTACATTG	GAAGATGCGGTCGATGACCA
CHRNB2	CAATGCTGACGGCATGTACGA	CACGAACGGAACTTCATGGTG
β-actin	CATGTACGTTGCTATCCAGGC	CTCCTTAATGTCACGCACGAT

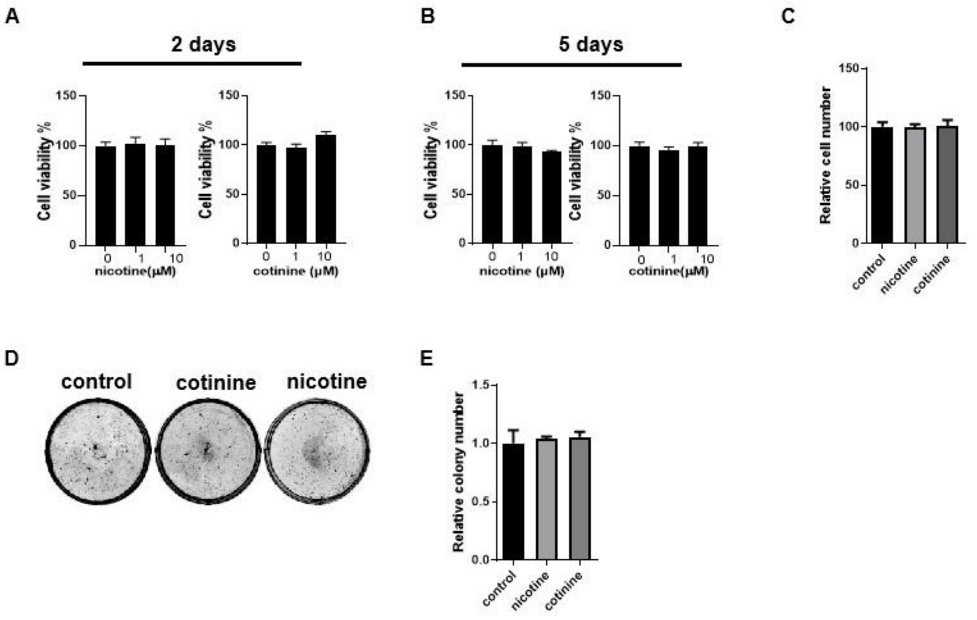

Figure S1. Nicotine or cotinine exposure has no effect on DMS53 cell proliferation. **A** and **B**) Cell titer blue assay results showed that nicotine or cotinine exposure for 2 days (**A**) or 5 days (**B**) has no effects on SCLC cell viability. **C**) Nicotine or cotinine exposure for 5 days has no influence on DMS53 cell proliferation. **D** and **E**) Colony formation analysis showed that nicotine or cotinine treatment has no effect on DMS53 cell colony number.

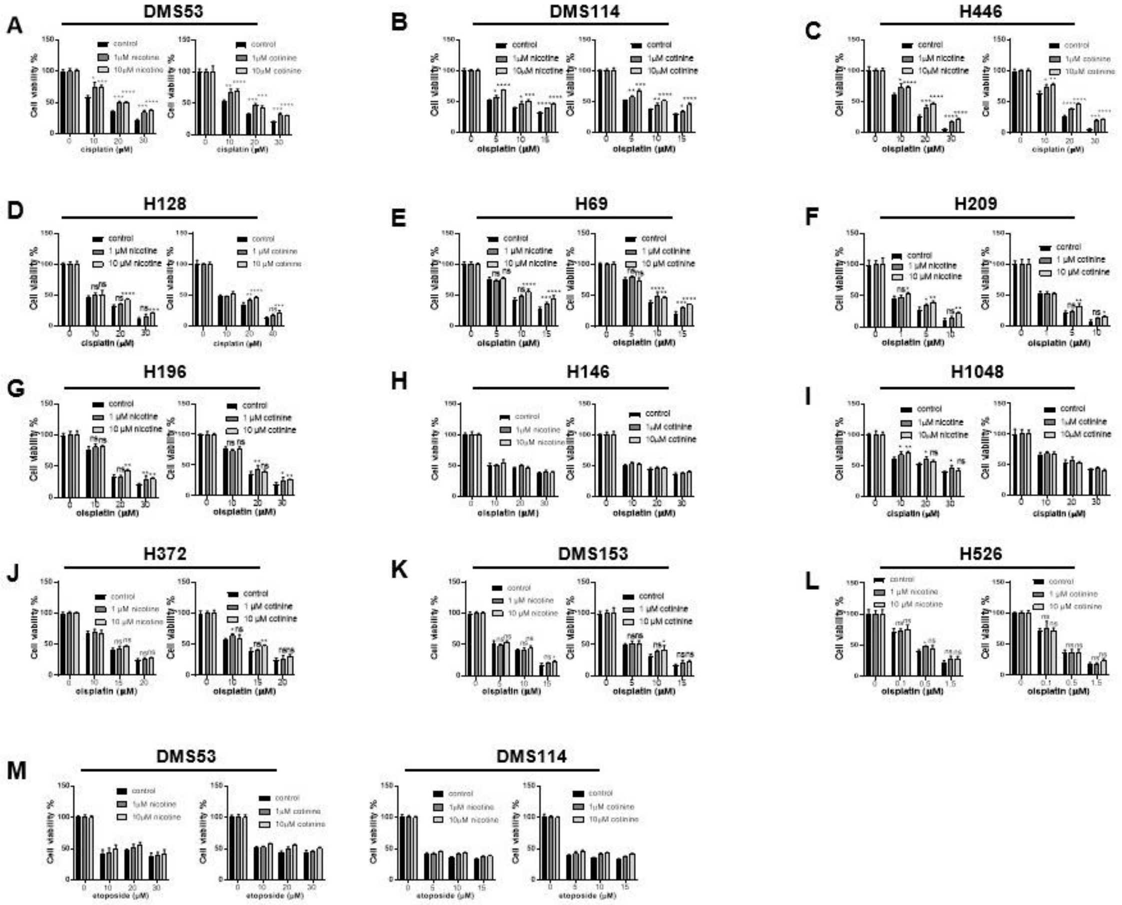

Figure S2. The effects of nicotine or cotinine exposure on the cytotoxicity of cisplatin and etoposide among different SCLC cells. **A**-**L**) DMS53, DMS114, H446, H128, H69, H209, H196, H146, H1048, H372, DMS153 and H526 cells were treated with cisplatin alone, or together with nicotine or cotinine and cell viability was measured by cell titer blue assay. **M**) DMS53 and DMS114 cells were treated with etoposide alone, or together with nicotine or cotinine and cell viability was measured. One-way analysis of variance (ANOVA) was used for data analysis. *, $P<0.05$; **, $P<0.01$; ***, $P<0.001$, ****$P<0.0001$.

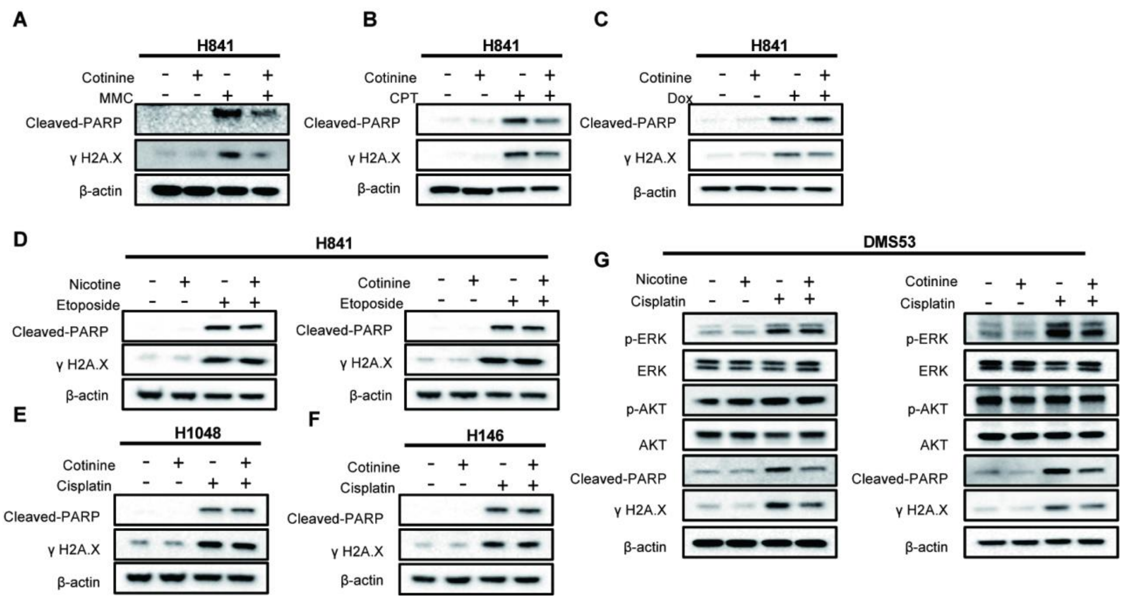

Figure S3. Effects of nicotine or cotinine on different chemotheraputic agents exposure among different SCLC cells with respect to DNA damage and cell apoptosis. **A**-**C**) H841 cells were treated with MMC (**A**), CPT (**B**) or Dox (**C**) alone, or together with cotinine and Western blot analysis was performed by using antibodies as indicated. **D**) H841 cells were treated with etoposide alone, or together with nicotine or cotinine and rH2A.X and cleaved-PARP were analyzed. **E**-**F**) H1048 and H146 were treated with cisplatin alone, or together with nicotine or cotinine and rH2A.X and Cleaved-PARP were analyzed. **G**) DMS53 cells were treated with cisplatin alone, or with nicotine or cotinine and Western blot analysis was performed by using antibodies as indicated. The whole blots could be found in the Figure S6.

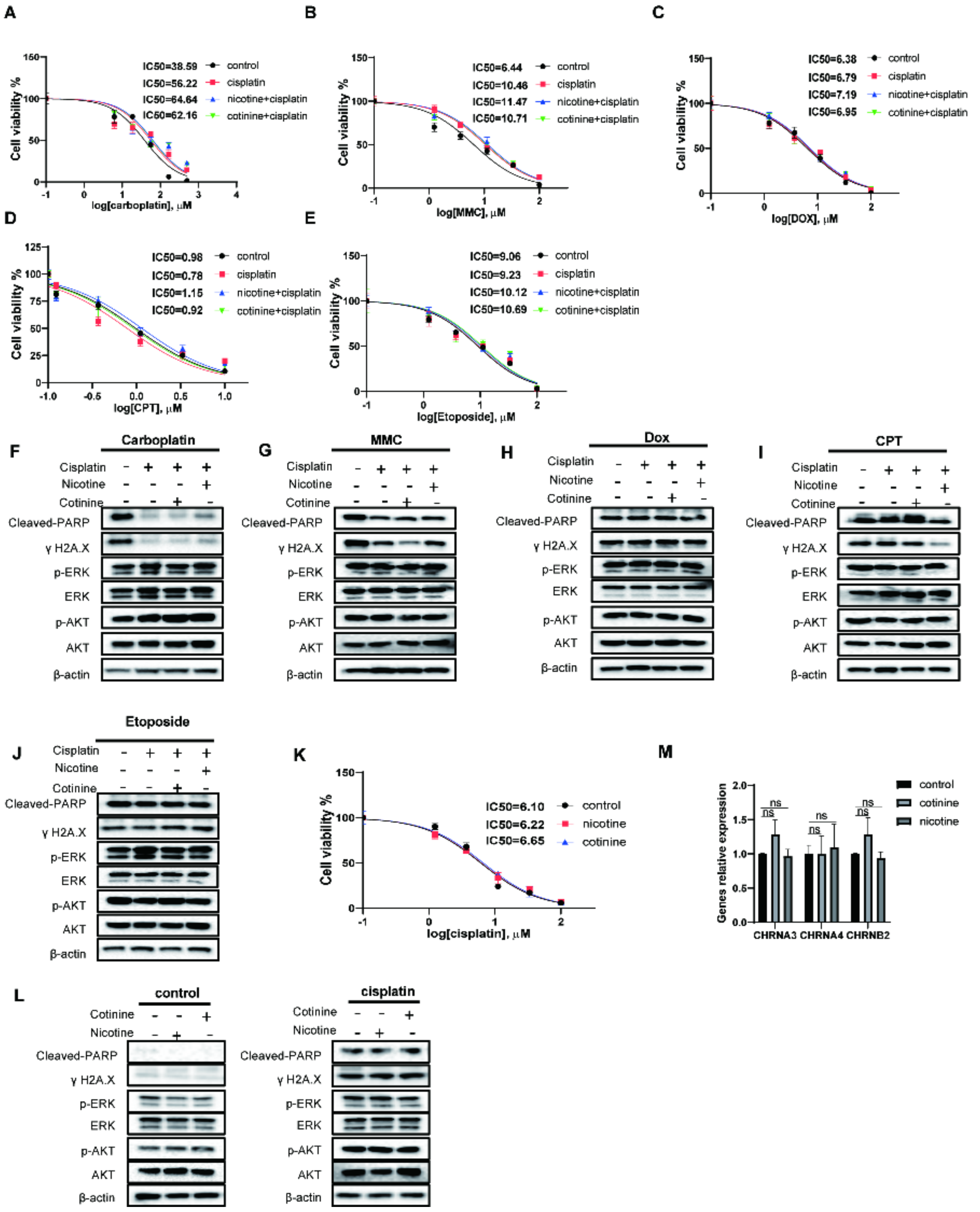

Figure S4. Cross-resistance of cisplatin-resistant H841 isogenic cell lines to other therapies. **A-E**, Four isogenic H841 cells with chronic exposure of cisplatin or in the presence of nicotine or cotinine cells were treated with carboplatin, MMC, DOX, CPT or etoposide for 48h with IC_{50}

measured by Cell Titer Blue assays. **F-J**, Western blot analysis of the four isogenic H841 cells upon the treatment of carboplatin, MMC, DOX, CPT or etoposide. **K** and **L**, Chronic exposure of nicotine or cotinine alone induced minimal resistance to cisplatin. **M**, Chronic exposure of nicotine or cotinine alone induced minimal up-regulation in CHRNA4, CHRNB2, and CHRNA3. The whole blots could be found in the Figure S6.

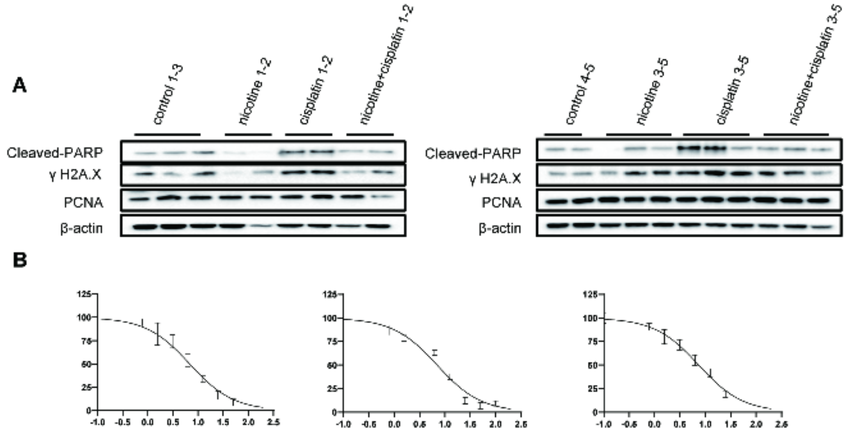

Figure S5. Nicotine enhances chemoresistance to cisplatin in a mouse xenograft model. **A**, Western blotting was used to determine the protein levels of cleaved-PARP, γ-H2AX, PCNA, and β-actin in the tumor samples from 5 individual mice in each group. **B**, Tumors from each group were digested into single cells (five tumors were combined in each group) and 20,000 cells were seeded into each well of 96-well plate. After 3-day cisplatin incubation, IC_{50}s of these cells to cisplatin were measured by Cell Titer Blue assay. The whole blots could be found in the Figure S6.

Figure 3A &3B

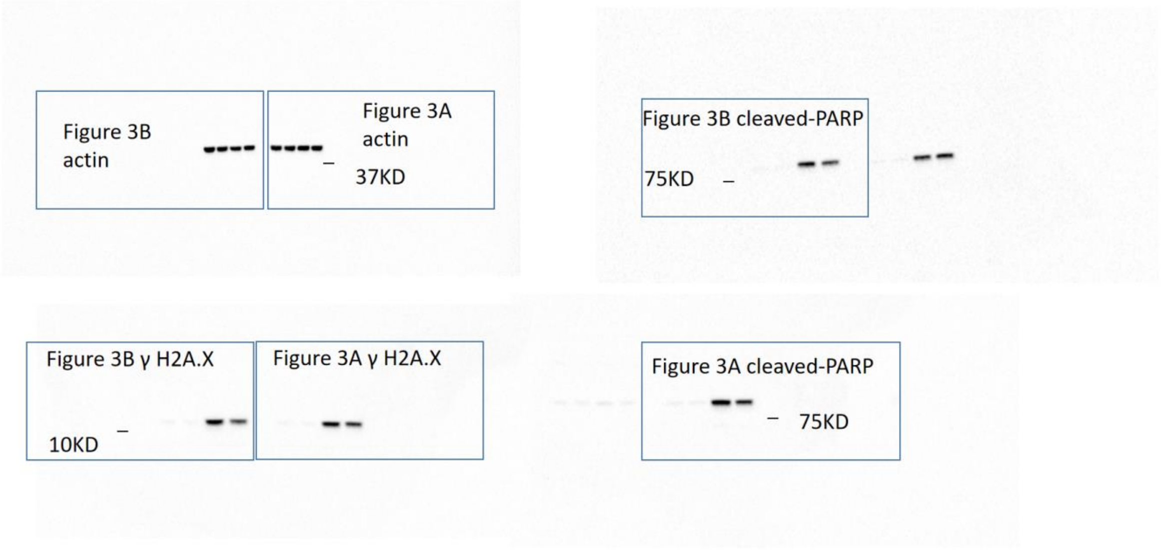

Figure 3 I

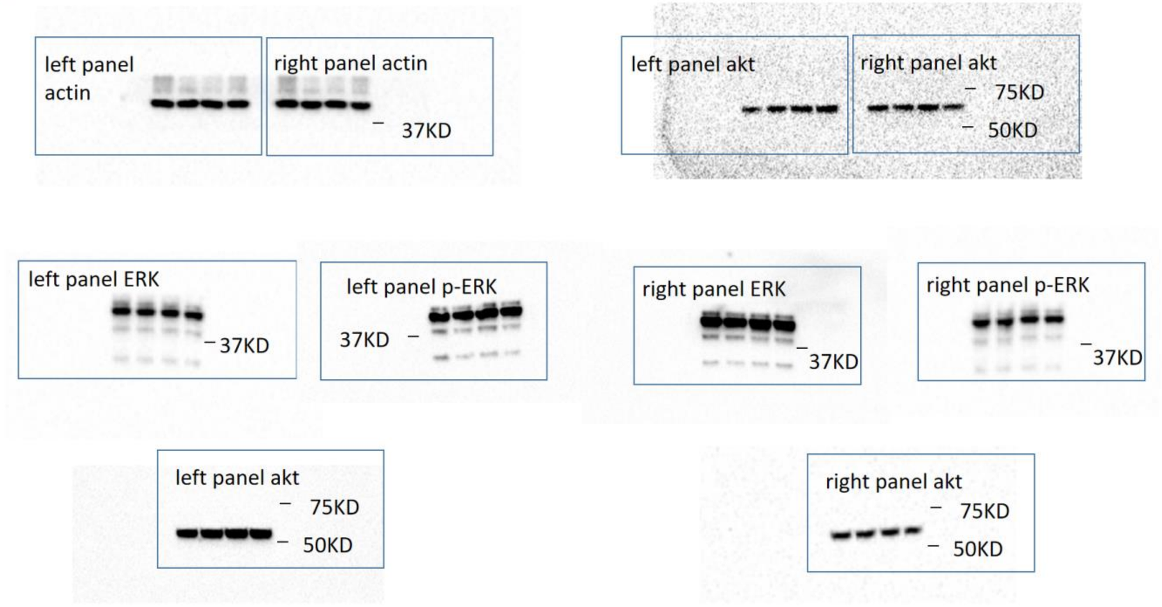

Figure 6C

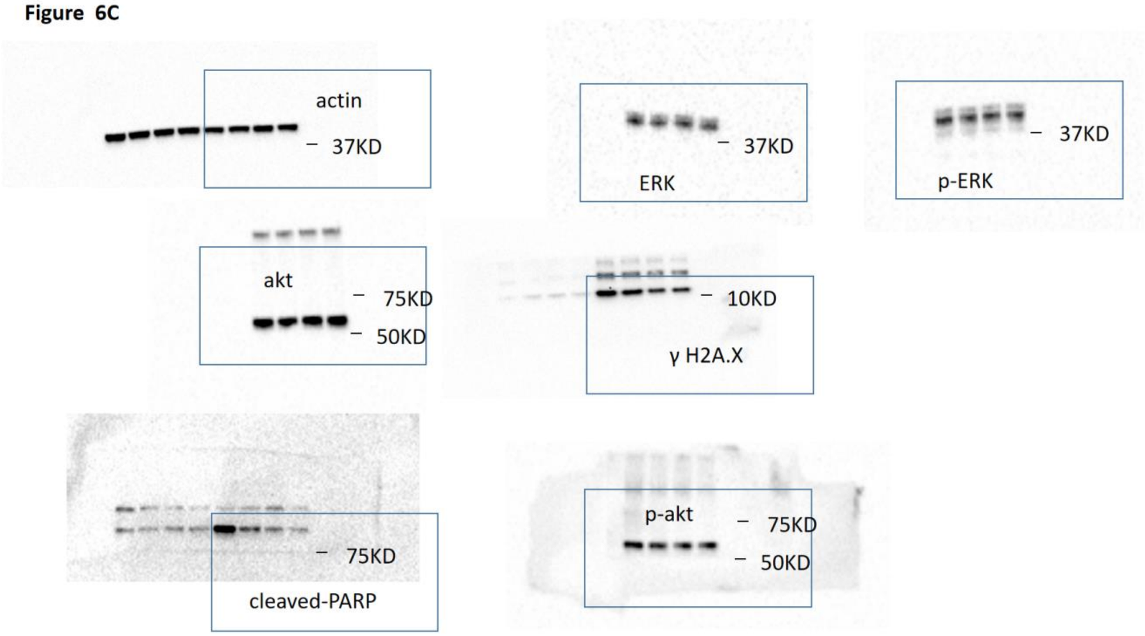

Figure 7E

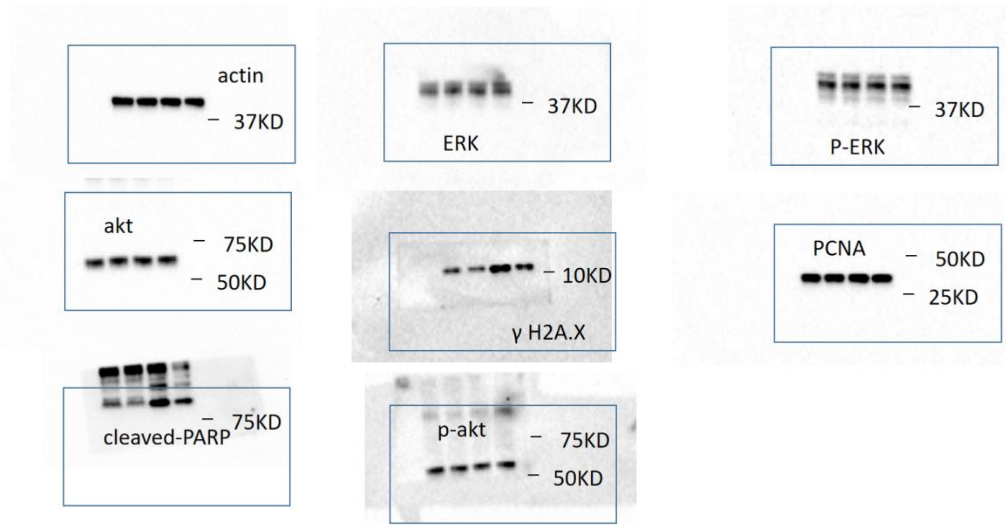

Figure S3

Figure S3A

Figure S3B

Figure S3C

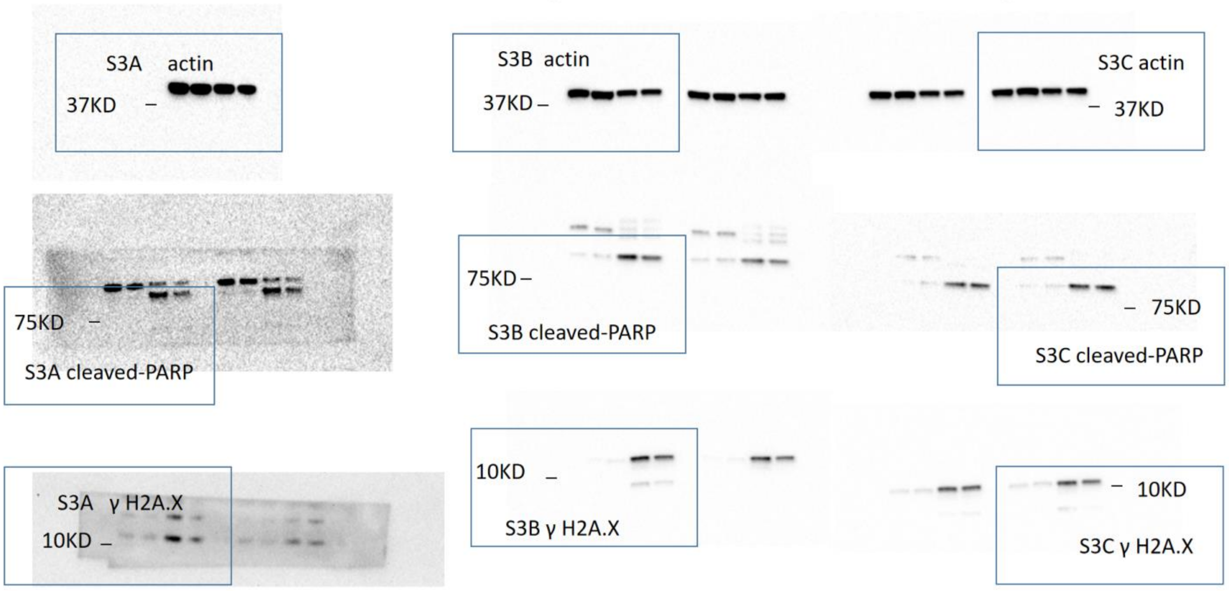

Figure S3

Figure S3D

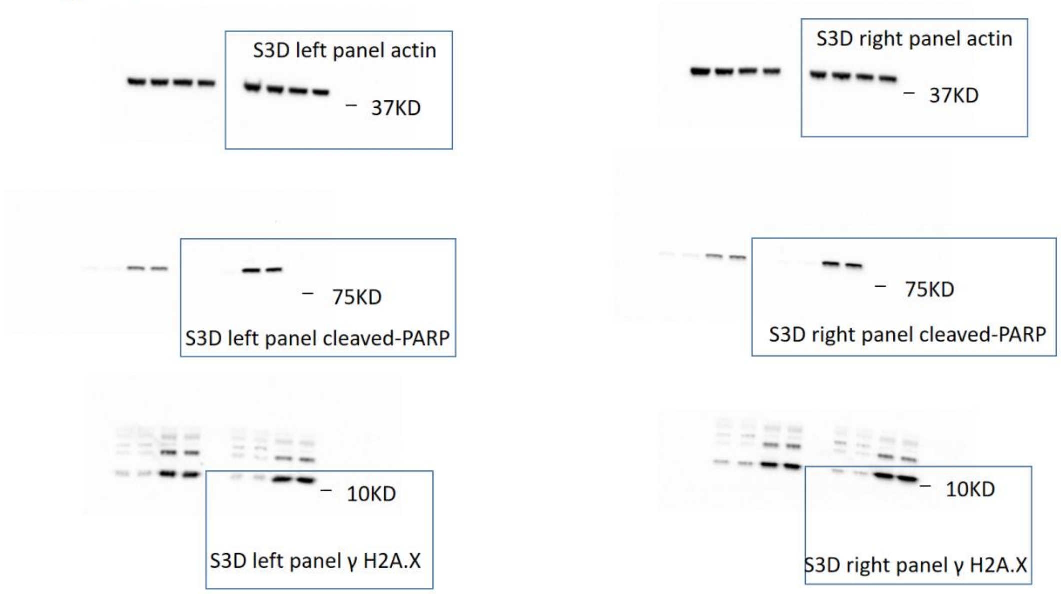

Figure S3

Figure S3E

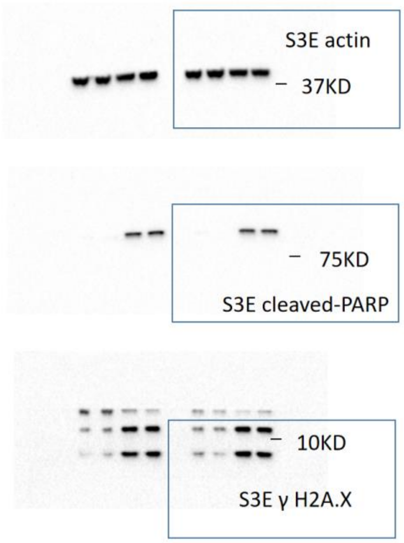

Figure S3F

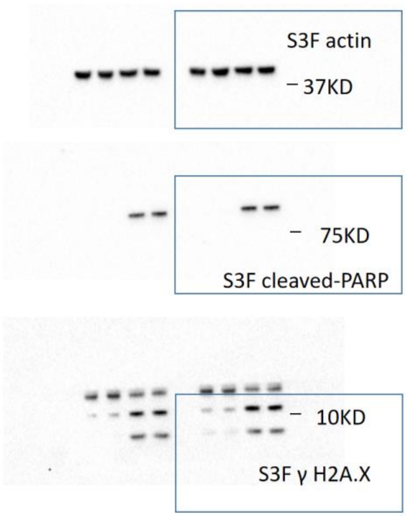

Figure S3

Figure S3G left panel

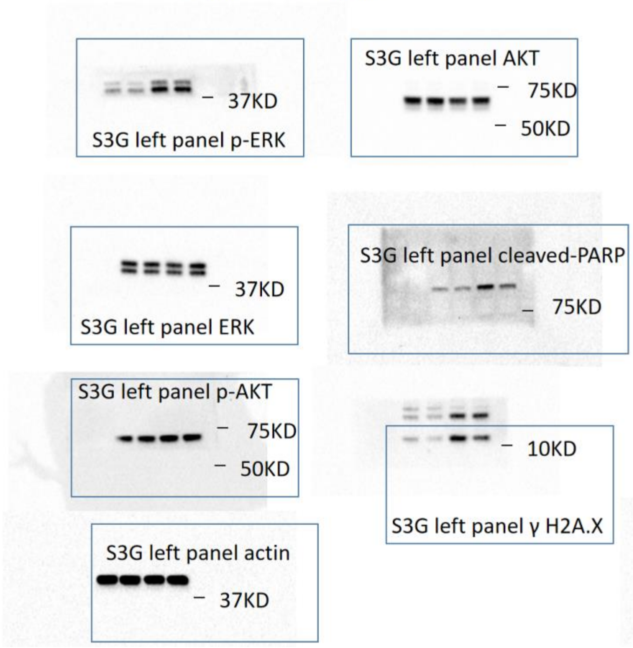

Figure S3G right panel

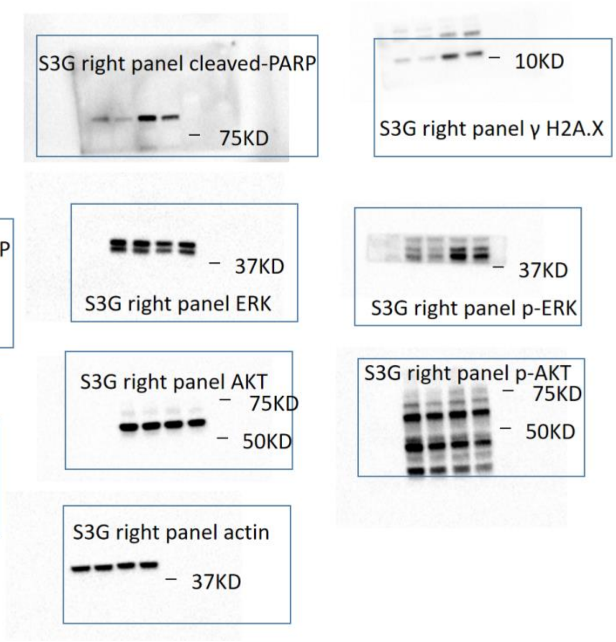

Figure S4

Figure S4F

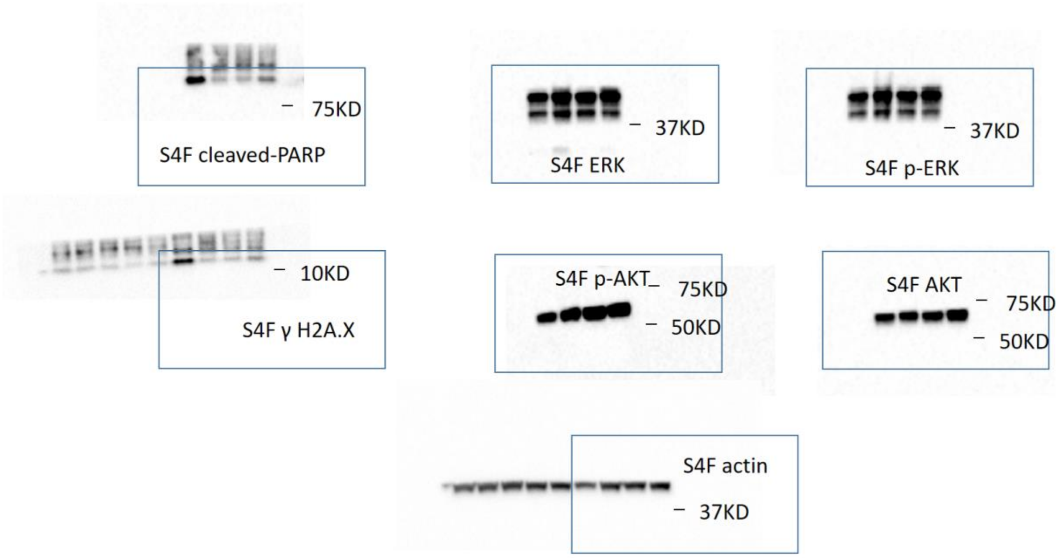

Figure S4

Figure S4G

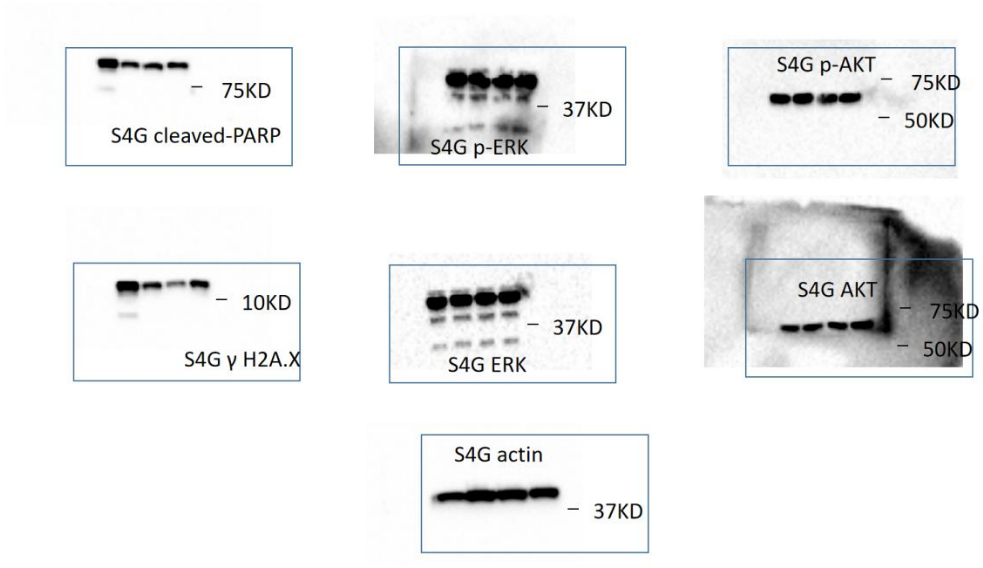

Figure S4

Figure S4H

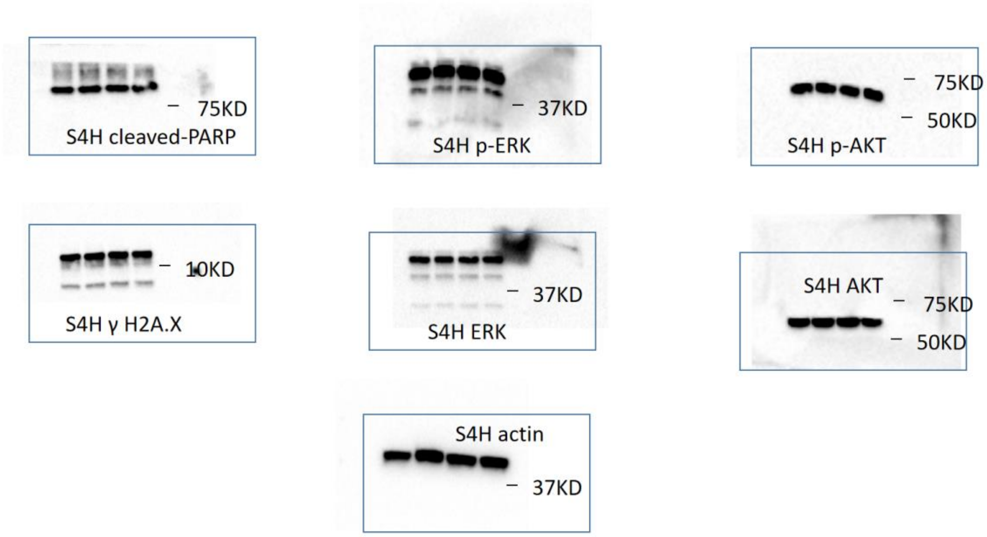

Figure S4

Figure S4I

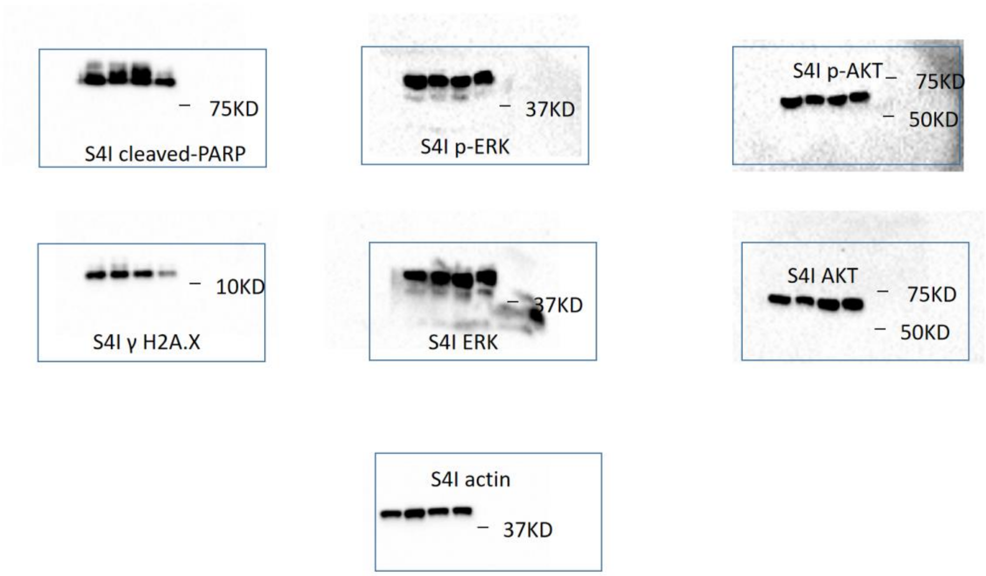

Figure S4

Figure S4J

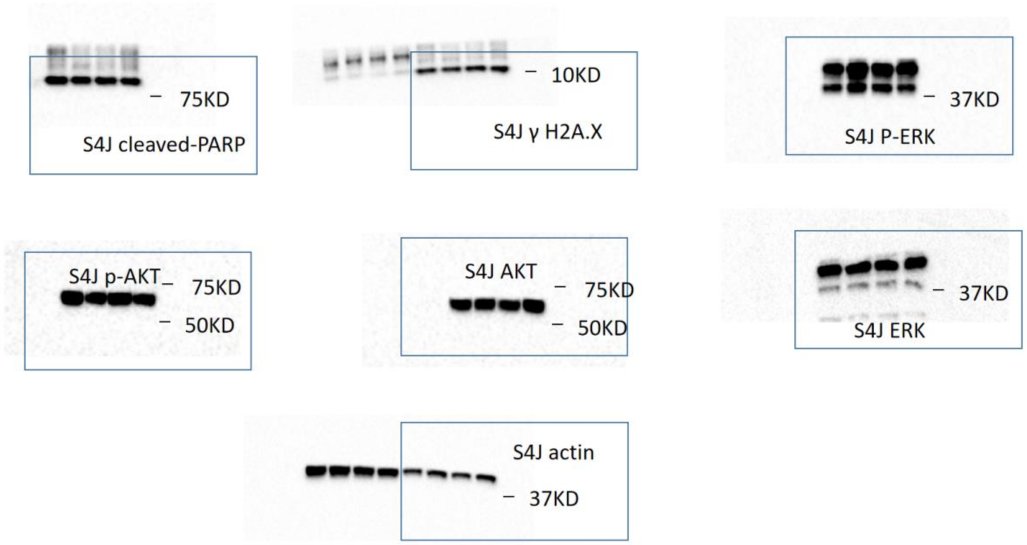

Figure S4

Figure S4L

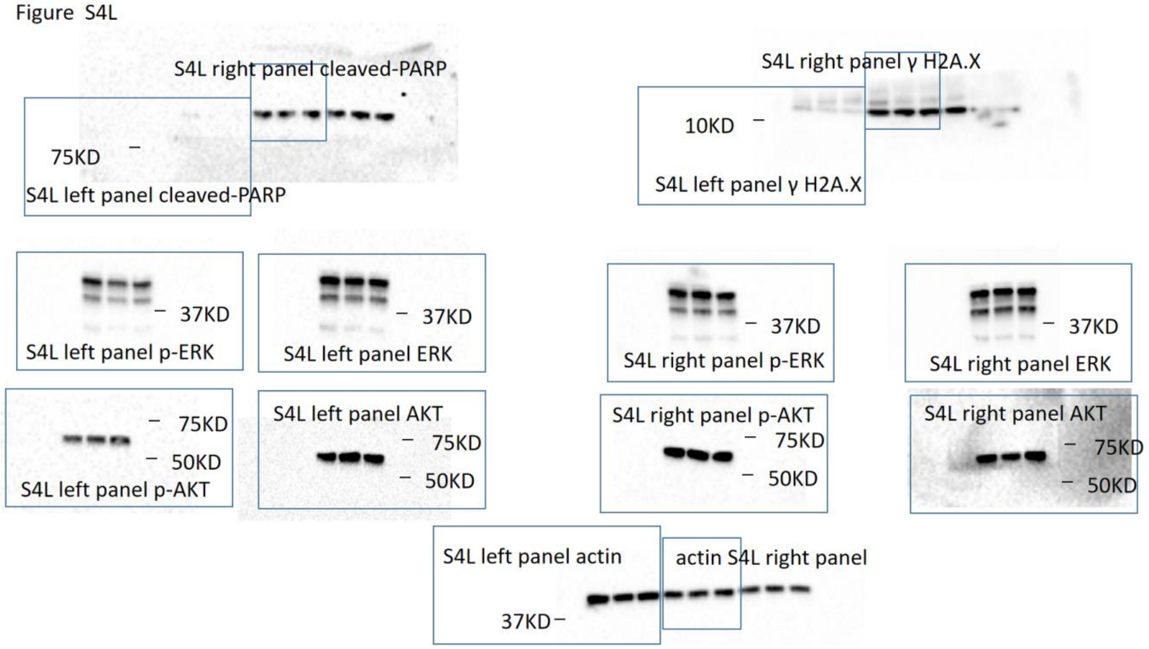

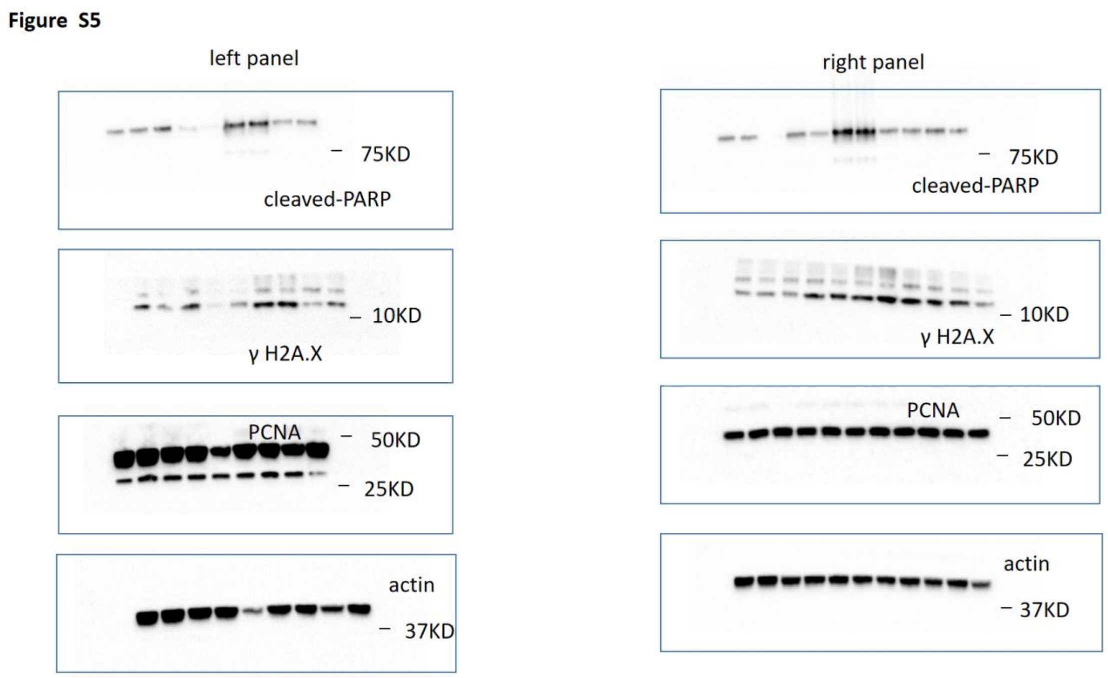

Figure S6. The whole blots.
